# First transcriptome profiling of *D*. *melanogaster* after development in a deep underground low radiation background laboratory

**DOI:** 10.1371/journal.pone.0255066

**Published:** 2021-08-05

**Authors:** Mikhail Zarubin, Albert Gangapshev, Yuri Gavriljuk, Vladimir Kazalov, Elena Kravchenko

**Affiliations:** 1 Joint Institute for Nuclear Research, DLNP, Dubna, Russia; 2 Institute for Nuclear Research, Russian Academy of Sciences, Moscow, Russia; Northwestern University Feinberg School of Medicine, UNITED STATES

## Abstract

Natural background radiation is a permanent multicomponent factor. It has an influence on biological organisms, but effects of its deprivation still remain unclear. The aim of our work was to study for the first time responses of *D*. *melanogaster* to conditions of the Deep Underground Low-Background Laboratory DULB-4900 (BNO, INR, RAS, Russia) at the transcriptome level by RNA-seq profiling. Overall 77 transcripts demonstrated differential abundance between flies exposed to low and natural background radiation. Enriched biological process functional categories were established for all genes with differential expression. The results showed down-regulation of primary metabolic processes and up-regulation of both the immune system process and the response to stimuli. The comparative analysis of our data and publicly available transcriptome data on *D*. *melanogaster* exposed to low and high doses of ionizing radiation did not reveal common DEGs in them. We hypothesize that the observed changes in gene expression can be explained by the influence of the underground conditions in DULB-4900, in particular, by the lack of stimuli. Thus, our study challenges the validity of the LNT model for the region of background radiation doses below a certain level (~16.4 nGy h^-1^) and the presence of a dose threshold for *D*. *melanogaster*.

## Introduction

All living organisms have been affected by natural background radiation since the time when life on the Earth began. The background radiation on the surface of the Earth consists of γ-rays, α- and β-particles, neutrons, radon, cosmic particles etc. from different terrestrial and space sources [1]. The dose rate of this background radiation varies in the range of 10^−7^–10^−5^ Gy h^-1^. Throughout life, all living organisms are constantly exposed to low doses of natural background radiation which induces generation of reactive oxygen species (ROS) and radicals in biological matter as well as destruction of biomolecules and cell structures. Therefore, ROS may be used as important signaling molecules or trigger stimuli in pathways supporting cell homeostasis [2–4]. The study of biological effects of background radiation reduction and determination of the value of very low-dose radiation for living organisms belong to a rapidly evolving field of interdisciplinary research which is focused on fundamental principles of the interaction between ionizing radiation and biological matter, damage-dose relations models, mechanisms of low-dose sensitivity, biological evolution and adaptation. The emphasis of such studies lies on the search for innovations which can be employed in astrobiology, cancer medicine and longevity research [3, 5–7].

Additionally, biological experiments in low-radiation background may prove or challenge the dose-damage linear no-threshold model (LNT) which linearly extrapolates effects induced by exposure of living organisms to high doses of radiation to the levels of low doses, natural background radiation and reduced background radiation. It should be noted that there are already a lot of data about the effect of low doses above the background radiation level, which allowed suggesting the hormesis theory that means possibility to reconsider the LNT model, a currently officially accepted, but actively debated model [1, 8–10]. However, available data on the effects of low doses below background radiation have not been sufficient yet to make a conclusion about the applicability of the LNT model to such conditions. Thus, studying responses of living objects to the low-radiation background environment gives the possibility to refine the LNT model in the low-dose region, to better assess radiation and cancer risks and to determine both evolutionary impact of natural radiation and mechanisms of radiosensitivity [3, 5].

Effects of background ionizing radiation deprivation on organisms have been studied for the last two decades [6, 11–17] in experiments performed in various locations, for instance, in the low-background chamber at Osaka University (Osaka, Japan) and several industrial underground tunnels, such as DUGL CJEM (Erdaogou Mine, China), BISAL (Boulby, UK) and WIPP (New Mexico, USA) [12, 14, 16, 18]. But most of biological studies dealt with deep underground laboratories, namely: LNGS (Gran Sasso, Italy), LSM CNRS (Modane, France), SNOLAB (Sudbury, Canada), JINR/BNO INR RAS (Neutrino village, Russia) [7, 11, 15, 17, 19–21]. They were specially designed for neutrino low-radiation background observatories and are the most efficient places for experimental isolation from cosmic and terrestrial sources of radiation. In order to develop effective shielding for low background laboratory it is necessary to take into account each component of the radiation background. For this reason, low-background laboratories are located deep underground, equipped with ventilation systems, additional chambers shielding from surrounding rocks and constructed from materials with a low-radionuclide content. All this makes it possible to reduce the total radiation background by 4–10 times [22, 23]. However, there are some limits to radiation background reduction in biological experiments. They concern inability to completely eliminate radioactive radon gas from laboratory atmosphere and ^40^K, a component of any nutrient media.

Briefly, the main scheme of biological experiments in deep underground low-background laboratories involves the study of two groups of identical model organisms simultaneously placed in conditions of low and natural background radiation. The exposition time is defined by purposes of research and by development/life cycle duration for multicellular organisms or generation time for microorganisms. The current results were obtained in such experiments when model organisms were exposed to low-background conditions from several days to about one year [11, 14, 20, 22]. Effects of low-radiation background on living organisms were determined by comparing experimental and control groups and by registering the changes in parameters suitable for experimental purposes, for instance, growth rate, lifespan, fertility, gene expression or protein abundance [12–15, 20]. An additional difficulty in carrying out such experiments is the demand to control many external parameters which must be the same for groups of organisms placed in low-background conditions and for those left in natural background conditions. These parameters are temperature, humidity, air pressure, gas composition, microparticles concentration and others since they can cause biological responses unrelated to the background radiation level.

At present the significant part of the effects registered in decreased background radiation conditions can be classified as suppressive, that reduce growth and development parameters, both for unicellular and multicellular organisms. Growth rate inhibition, increased sensitivity to radiation, delayed recovery after return to natural background conditions, alteration in expression of several genes concerned with ribosomal proteins, membrane transport, respiration, reparation and antioxidant regulation for increased ROS removal were observed in experiments on mammalian cell cultures (TK6, V79, M10, L5178Y, FD-LSC-1) and unicellular organisms (*Paramecium tetraurelia*, *Synechococcus lividus*, *Shewanella oneidensis*, *Deinococcus radiodiodurans*, *Saccharomyces cerevisiae*) [11, 12, 14, 17, 18, 24, 25]. The first multicellular organism used for determination of the effects of low-radiation background was the fruit fly in projects FLYINGLOW and RENOIR [20, 26]. These experiments established that *D*. *melanogaster* was well suited for maintenance in environments of a deep underground laboratory. During several weeks of exposition to low-background conditions, this model organism demonstrated the increased median lifespan for both sexes and fertility reduced by 30% [20]. After eight months of exposure of nematode *Caenorhabditis elegans* to low-radiation background the increased egg-laying and relatively faster growth rates during larval development were registered, and after 72 hours in an underground laboratory alterations in expression of genes involved in spermatogenesis (up-regulated), collagen and cuticle metabolism (down-regulated) were observed [22]. During the ongoing REPAIR project the lake whitefish *Coregonus clupeaformis* was used in SNOLAB where a significant increase in embryo body length and body weight was registered, but the authors could not explain the reason for these changes [27].

The explanation of stress-like responses in conditions of low-radiation background is commonly based on the hypothesis that decrease in radiation background leads to reduction of ionization events at the cellular level, which, in turn, affects production and removal of ROS, being signaling molecules and trigger stimuli to several processes, for instance, the DNA reparation process [3, 6, 14, 21]. In other words, it is assumed that the impact of natural background radiation may have a stimulatory effect and therefore is useful for life. However, no effects of decreased background radiation were revealed for *Escherichia coli* and *Bacillus subtilis* in several experiments at Modane and Boulby [13, 16, 21]. In controlled evolution experiments during two weeks 500 generations of *E*. *coli* developed in low-radiation background and no differences in evolutionary trajectories were found compared with bacteria developed in the natural background [13]. This result was supported by GEANT4-DNA modeling based on Monte Carlo method that did not confirm the hypothesis that natural background radiation may have significant influence on ROS production in unicellular organism. According to the modeling, less than one cell out of 10000 per day interacts with components of terrestrial natural radiation background [21]. Therefore, it is highly unlikely that background ionizing radiation may have any perceptible influence on cell homeostasis [21]. Another experiment at the Boulby laboratory demonstrated the same growth rate of *E*. *coli* and *B*. *subtilis* in low-background and natural background conditions, which makes it possible to assume the existence of a threshold in the radiation dose below which the LNT model cannot be applied and the absence of hormetic effects in the range of natural radiation background for bacteria [16].

It is important to note that many researchers who have conducted biological experiments in low-background laboratories, for instance, SNOLAB and DUGL CJEM, point out that some factors, not related to radiation, influence biological responses, which further complicates the analysis of the obtained data [12, 15].

To summarize, the explanation of low background radiation effects on living organisms may widen our knowledge about mechanisms of radiosensitivity, hormesis processes, signaling role of ROS and make possible to improve radiation risk model in the range of natural background and low doses of ionizing radiation [7, 16, 28, 29].

In our work we study the effect of low-background radiation on an important model organism *D*. *melanogaster*. The flies, from larva to imago, were exposed in the Deep Underground Low-Background Laboratory DULB-4900 (BNO INR RAS, Russia) during 14 days that, as reported earlier, is an appropriate exposure period to observe effects of reduced background radiation [20]. We aim to register for the first time the response of this complex multicellular organism to reduced environmental radiation at the whole transcriptome level using RNA-seq profiling and to analyze the obtained results in terms of the impact of different types of stress including radiation treatment. This study affords to estimate the obtained results from the point of view of the LNT model in the low-dose range and to widen knowledge about the influence of deep underground conditions on living organisms. Also, we declare our work to be the first initiative of interdisciplinary studies in the BNO (INR RAS) facility which links tasks of biophysics, radiobiology, astrobiology and medicine. The unique DULB-4900 laboratory has a high potential for hosting biological experiments [7].

## Materials and methods

### *D*. *melanogaster* stocks and maintenance

We used wild-type *D*. *melanogaster* strain Oregon-R cultured on a medium containing 1000 mL water, 16 g yeast, 30 g sugar, 40 g semolina, 7 g agar and 0.5 mL propionic acid at 24°C in dark. The experiments were simultaneously carried out in a chamber of the low-background underground laboratory DULB-4900 in the horizontal tunnel of the Baksan Neutrino Observatory (BNO) equipped with temperature control and ventilation systems, and in the laboratory of the BNO ground institute building located near the entrance of the tunnel where the control biological group was maintained in natural background conditions. During one experiment, twenty Oregon-R males and females were placed in each laboratory in vials with the medium for egg-laying and were discarded after 48 hours, then three vials were kept in DULB-4900 and two vials in the ground laboratory for the entire fruit fly development cycle (14 days) before getting 2–3 days adult flies.

### RNA extraction, library preparation and sequencing

Twenty five 2–3 days adult males from each vial from DULB-4900 and the ground laboratory were homogenized in RNA-intact (Evrogen, Russia) and transferred on the ice to the Evrogen company (Moscow, Russia). Total RNA was isolated from these samples using ExtractRNA reagent (Evrogen, Russia) according to the manufacturer’s standard protocol. The quality of total RNA was verified by gel electrophoresis using ~1 μg of extracted RNA (S1 Fig). Using the TruSeq mRNA Stranded reagent kit (Illumina, USA) poly (A+) fractions of total RNA were enriched and then cDNA was synthesized by random hexamer priming. The resulting cDNA was used to prepare libraries compatible with Illumina sequencing technology in the Evrogen company. The quality of the resulting libraries was determined using the Fragment Analyzer system (Agilent, USA). The quantitative analysis was performed by qPCR. After the quality control and DNA quantity estimation the library pool was sequenced with 100 bp single-end reads on Illumina NovaSeq 6000 platform (Illumina, USA). FASTQ files were obtained using bcl2fastq v2.20 Conversion Software (Illumina, USA). As a result, 556 979 047 reads were received.

### RNA sequencing data analysis

The samples were analyzed as two comparison groups with three biological repeats within the “LB” (low background) group and two biological repeats within the “NB” (control, natural background) group (S2 Fig). Quality control of sequencing results performed in the FastQC 0.11.9 program, showed high quality of readings and the presence of a small number of adapter sequences in them. Preliminary filtering of readings by length and quality, as well as the removal of adapter sequences, were carried out using TrimGalore 0.6.1 and Cutadapt 2.10. As a result, more than 98% of the data passed pre-filtering and was used in the subsequent analysis. The assembly of BDGP version 6 (BDGP Release 6 + ISO1 MT/dm6, source of UCSC) with masked repeating elements was used as the reference genome of *Drosophila melanogaster* to focused solely on sequences of known annotated genes that most likely do not contain repetitive elements (the limitation of this approach does not take into account the expression of transposons). The readings were mapped to the reference genome using HiSat2 2.2.1 taking into account splicing sites and exon boundaries according to the NCBI RefSeq annotation. Evaluation of the quality of the mapping results was carried out using the RSeQC v3.0.1 package.

More than 93% of the readings were mapped for each of the samples while the percentage of the readings mapped to exons was 73–76%. The difference in gene expressions was calculated using the HTSeq 0.13.5 software. The resulting expression matrices were analyzed in R 3.6.2 medium using the DESeq2 1.30.1 library. The genes with less than 10 readings were not used in the analysis. Raw and processing sequencing data were published in NCBI GEO database via accession number GSE159477.

### Functional annotation, biological networks, Venn diagrams and NASA GeneLab Data

Biological process term enrichment analysis and KEGG pathway ontology were performed with DAVID (version 6.8). DAVID recommended defaults were used for all statistical parameters for defining annotation clusters. Biological process networks for differentially expressed genes were constructed with Cytoscape 3.8.0 Bingo 3.0.4 tool [30]. Venn diagrams representing the quantity of shared genes were made with Funrich 3.1.3 [31].

GeneLab data (https://genelab-data.ndc.nasa.gov/genelab/accession/GLDS-278/) are courtesy of the NASA GeneLab Data Repository (https://genelab-data.ndc.nasa.gov/genelab/projects/).

### RT-qPCR

The difference in the expression level for several genes from different functional categories obtained with RNA-seq analisis was verified by RT-qPCR. RNA was treated with dsDNase and converted into cDNA using Maxima First Strand cDNA Synthesis Kit for RT-qPCR with dsDNase (Thermo Fisher Scientific) according to the manufacturer’s recommended procedures. Quantitative RT-PCR was carried out using iTaq Universal SYBR Green Supermix (BioRad) on a CFX96 Touch Real-Time PCR Detection System (BioRad). The qPCR reactions were performed in triplicate. RT-qPCR data were obtained with the ΔΔCt method using normalization to the reference gene *RpL32*. The primer sequences are listed in the S1 Table.

### Measurement of radiation purity (contamination) of medium

To assess the radiation purity (contamination) of medium fractions of isotope decays with gamma rays data output was measured using the low-background gamma-ray spectrometer SNEG (BNO INR RAS, Russia). Two samples of the medium weighing ~70 g were measured during 158 and 180 hours. The concentrations of ^226^Ra, ^232^Th, ^208^Tl and ^40^K were found in both measurements according to the size of the 609 keV, 911 keV, 2615 keV and 1460 keV gamma-ray peaks in the spectrum with the correction for the detector background.

### Experimental properties both DULB-4900 (Deep Underground Low-Background Laboratory) and the laboratory in the ground institute building BNO (INR RAS)

**Fig 1:**
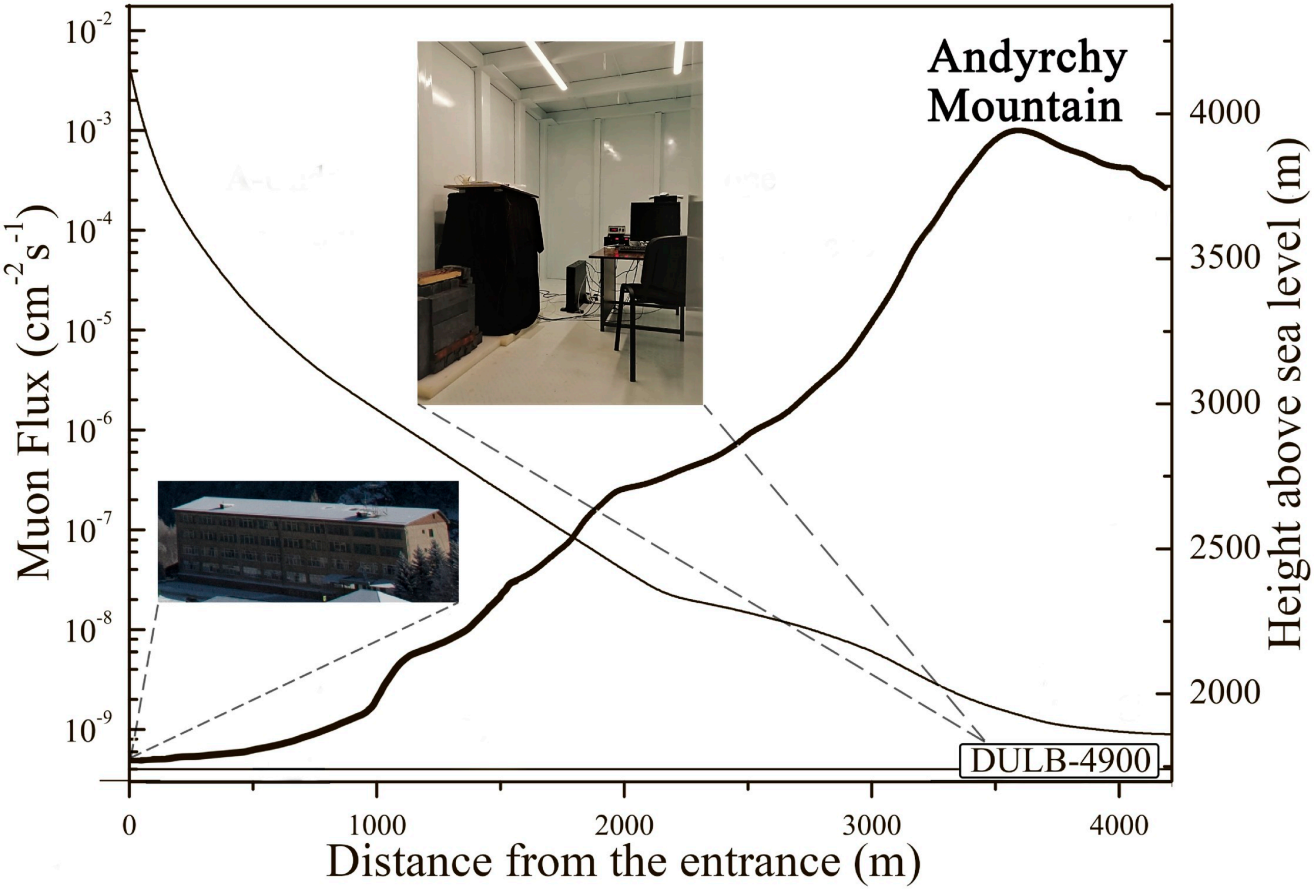
Locations and muon flux parameters of the BNO ground laboratory and the DULB-4900 low-background laboratory in the Andyrchy mountain (data from [32]).

Our experiment was performed in the low-background laboratory DULB-4900 of the Baksan Neutrino Observatory of the Institute for Nuclear Research of the Russian Academy of Sciences (BNO INR RAS / Neutrino village, North Caucasus, Russia) which has unique shielding properties [7, 19, 32]. It is located in the farthest part (3700 meters from the entrance) of the horizontal tunnel in the Andyrchy mountain at the depth of 1800 meters under the rock mass (4900 m.w.e.) (Fig 1). To efficiently shield the area against surrounding rock mass radiation and to provide stable experimental conditions the underground laboratory is equipped with special chambers with individual multilayer shielding consisting from 20 cm of polyethylene, 2 mm of cadmium and 15 cm of lead (from outer layer to inner one) [32]. Cosmic-ray flux deprivation in DULB-4900 results in residual values: the muon flux is 3.0 × 10^−9^ sm^-2^s^-1^ (NB– 2.0 × 10^−2^) (Fig 3), the neutron flux is less than 3.8 × 10^−7^ cm^-2^s^-1^ (NB—4.67 × 10^−3^), the gamma-ray flux is 0.02 nGy h^-1^ (NB– 120 nGy h^-1^). Due to the special ventilation system and implementation of constructing materials with a low content of radionuclides, the radon activity in the underground laboratory chamber is 25.2 Bq m^-3^ (NB– 35 Bq m^-3^) and it stays near this level throughout the year [32–34].

Not only monitoring of muon and neutron fluxes, gamma-ray background and radon concentration but also the control of non-radiation parameters, such as temperature, atmospheric pressure and gas composition in the laboratory are important for biological experiments. Atmospheric pressure in DULB-4900 and in the ground laboratory was about 620 mmHg, temperature was stabilized at 24°C, gas composition underground was maintained with the general tunnel ventilation system and the local low-background chamber ventilation system [32]. In the reference ground laboratory there were no physical installations or significant physical equipment that could influence the experiment.

## Results

### Estimation of the total background radiation in DULB-4900 and in the laboratory of the ground institute building of BNO (INR, RAS)

As it has been already demonstrated for biological experiments in deep underground low-background laboratories [11, 21, 23] exceptional shielding of such laboratories reduce the muon, neutron and gamma fluxes by ~10^3^ times (Table 1) and some components may be assumed to be negligible in our calculations of total background radiation. The rate of reduction is so high that ^40^K and radon are the main contributors to the total background in biological studies [23] in low-background laboratories.

**Table 1:** Components of radiation background in DULB-4900 and the ground laboratory in the institute building of BNO (INR, RAS).

Background component	Data source	Ground laboratory in the institute building, BNO (INR, RAS)	Chamber of DULB-4900, BNO (INR, RAS)
Gamma, nGy h^-1^	NaI(Tl) crystal scintillation detector [32]	120	0.02
Neutrons, nGy h^-1^ (cm^-2^s^-1^)	Helium proportional counter [32, 33]	3.45 (4.67 × 10^−3^)	~0 (3.8 × 10^−7^)
Muons and cosmic rays, nGy h^-1^ (cm^-2^s^-1^)	Determined by the altitude (m.a.s.l.) and covering rock massive (m.w.e) [32]	24.4 (2.0 × 10^−2^)	~0 (3.0 × 10^−9^)
Radon, nGy h^-1^ (Bq m^-3^)	Experimental set-up to continuously measuring the radon activity [33, 34]	1.19 (35)	0.85 (25)
Nutrition medium ^40^K, nGy h^-1^ (Bq kg^-1^)	Spectrometer SNEG	15.5 (6.7)	15.5 (6.7)
Total dose rate, nGy h^-1^	Estimation	164.5 (190.7—based on UNSCEAR data)	16.4

The impact of the radon component on natural background is a highly debated issue of radiobiological studies, and the rate of exposure to α-particles being emitted by radon gas is defined by the level of organization of a model organism used in experiments, its body surface area, its type of physiology and respiratory system [11, 21, 35, 36]. The levels of radon, 25 Bq m^-3^ in DULB-4900 and 35 Bq m^-3^ in the BNO ground laboratory, are significantly lower than the world average radon level of 50–100 Bq m^-3^ [1] and at the same time they are similar to those of previous experiments on multicellular organisms in this field [20, 22]. The complete elimination of the radon component from natural background radiation can be achieved in the future with special experimental setups for deep underground biological studies such as low radon cleanrooms with special air filtration systems etc. [37].

Since naturally occurring radioisotopes, one of the constant sources of background radiation, cannot be shielded, we estimated radiochemical purity (contamination) of the nutrient medium for *D*. *melanogaster*. The activity of ^226^Ra was 0.037 ± 0.017 Bq kg^-1^, of ^232^Th– 0.042 ± 0.007 Bq kg^-1^, of ^208^Tl– 0.055 ± 0.021 Bq kg^-1^, significantly lower than the impact of ^40^K isotope– 6.66 ± 1.82 Bq kg^-1^. It is interesting that the sum of these values (~6.8 Bq kg^-1^) is quietly lower than estimations of many other nutrition components which vary in range 40–600 Bq kg^-1^ [38] and it is similar to some measurements in the field of low-radiation background biology [23].

According to the protocols of total natural background estimation [11, 21–23, 39] we obtained resulting natural background radiation in the chambers of DULB-4900 at the level of ~16.4 nGy h^-1^ and the total background radiation in the ground laboratory in the institute building of BNO (INR, RAS) at the level of ~190 nGy h^-1^ (Table 1). Due to the high-altitude location of BNO (INR, RAS) 1670 meters above sea level an elevated impact of the cosmic component to the total radiation background was observed [1, 40]. The difference between natural background and low-background radiation in DULB-4900 indicates ~10-fold background reduction, which means the appropriate level for low-radiation background biological studies in DULB-4900 [23].

### Differential gene expression in *D*. *melanogaster* developed in DULB-4900 and in the BNO ground laboratory

During our experiment we studied differentially expressed genes between synchronized flies from groups exposed to natural and low radiation background. To identify such genes we carried out an RNA-seq analysis of three prepared separately repeats of 25 males from flies developed in DULB-4900 (LB-flies (low background)) and two prepared separately repeats of 25 males from flies developed in the ground laboratory (NB-flies (natural background)). Only 77 (0.44%) transcripts representing 76 genes had different abundance (more than 1.5-fold, FDR<0.05) between LB-flies and NB-flies. 31 genes (40%) were up-regulated and 45 genes (60%) were down-regulated in LB-flies vs. NB-flies (Table 2).

**Table 2:** Differentially expressed genes of the LB-flies vs. the NB-flies and corresponding biological processes or an activity based on the FlyBase annotation (FB2020_04 release). FDR<0.05, LFC—Log_2_ fold change.

LFC	Gene Symbol	Biological Process or Activity	LFC	Gene Symbol	Biological Process or Activity
4.31	CG33462	Proteolysis	-0.78	Hex-C	Glucose homeostasis
3.96	lncRNA:CR32865	Unknown function	-0.80	Shmt	Regulation of circadian rhythm
3.68	CG14205	Predicted transferase activity	-0.81	AdSL	Predicted ’de novo’ AMP biosynthetic process
3.08	CG14219	Predicted transferase activity	-0.86	CG14400	Unknown function
2.90	CG10182	Predicted transferase activity	-0.87	ry	Xanthine dehydrogenase activity
2.74	GNBP-like3	Defense response to other organism	-0.88	Vmat	Neurotransmitter transport
2.65	IM23	Antibacterial humoral response	-0.90	mino	PiRNA metabolic process
2.25	Drs	Defense response	-0.94	tutl	Mechanosensory behavior
2.20	Fst	Cold acclimation	-0.94	Paics	’De novo’ IMP biosynthetic process
2.12	IM1	Defense response	-0.95	CG33080	Predicted carbohydrate metabolic process
2.09	MtnD	Metal ion homeostasis	-0.95	CG12766	Predicted oxidation-reduction process
2.09	CG13215	Unknown function	-0.96	CG10960	Predicted transmembrane transport
2.08	CG14957	Predicted chitin binding activity	-1.01	Nep6	Predicted proteolysis
2.02	CG33470	Unknown function	-1.04	Sardh	Sarcosine catabolic process
1.99	CG10337	Oxidoreductase activity	-1.04	OtopLa	Unknown function
1.99	CG13075	Predicted chitin binding activity	-1.06	Shmt	Regulation of circadian rhythm
1.94	Spn88Eb	Predictede endopeptidase inhibitor activity	-1.09	lectin-28C	Predicted galactose binding activity
1.88	Tig	Cell adhesion mediated by integrin	-1.11	CG15534	Predicted sphingomyelin catabolic process
1.72	CG43773	Unknown function	-1.13	Spat	Glyoxylate catabolic process
1.68	Tep2	Innate immune response	-1.15	CG43055	Predicted galactose binding activity
1.64	CG15065	Predicted defense response	-1.17	CG4716	Methylenetetrahydrofolate dehydrogenase [NAD(P)+] activity
1.61	IM3	Antibacterial humoral response	-1.22	CG7542	Predicted proteolysis
1.57	IM2	Defense response	-1.31	CG8834	Predicted fatty acid biosynthetic process
1.48	Mat	Unknown function	-1.37	CG31778	Predicted to have serine-type endopeptidase inhibitor activity
1.47	Tep1	Innate immune response	-1.41	Ser8	Predicted proteolysis
1.45	CG13324	Unknown function	-1.49	hll	Regulation of circadian sleep/wake cycle, sleep
1.21	CG18609	Predicted fatty acid elongation	-1.72	CG14120	Predicted endoribonuclease activity
0.89	CG34198	Unknown function	-1.87	CG33511	Unknown function
0.85	lncRNA:CR44493	Unknown function	-1.87	CG31089	Predicted lipid metabolic process
0.69	Mst84Dc	Sperm axoneme assembly	-1.96	CG18179	Predicted proteolysis
0.63	Tsp42Ec	Unknown function	-1.98	CG34316	Unknown function
-0.59	su(r)	’De novo’ pyrimidine nucleobase biosynthetic process	-2.05	CG34136	Unknown function
-0.64	alpha-Est1	Carboxylesterase activity	-2.07	CG18540	Unkown function
-0.70	Pect	Ethanolamine-phosphate cytidylyltransferase activity	-2.16	asRNA:CR45604	Unknown function
-0.71	CG2233	Unknown function	-2.23	LManVI	Predicted mannose metabolic process
-0.73	Nepl9	Predicted proteolysis	-2.32	CG15533	Predicted sphingomyelin catabolic process
-0.73	Odc2	Predicted putrescine biosynthetic process from ornithine	-3.09	LManIV	Predicted mannose metabolic process
-0.74	Cyt-b5-r	Predicted lipid metabolic process	-5.17	up	Muscle contraction, myofibril assembly
-0.76	nAChRbeta1	Synaptic transmission			

**Fig 2:**
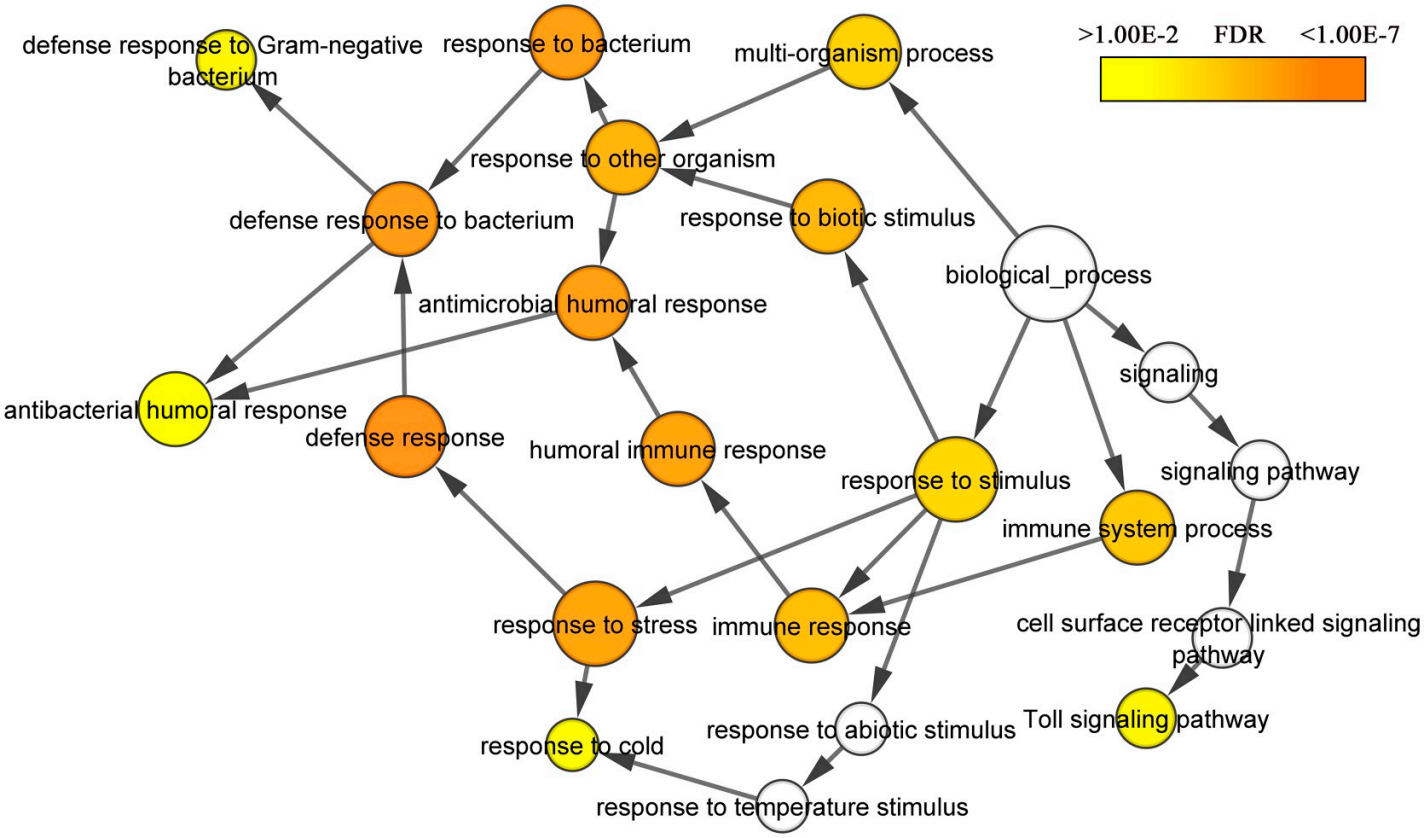
Gene ontology biological process term enrichment analysis for up-regulated LB-flies genes.

We used the DAVID analysis (version 6.8) for differentially expressed genes to identify significantly enriched GO terms in the “Biological process” category. Six terms were overrepresented among up-regulated in LB-flies genes with p-value < 0.05: defense response (6 genes), innate immune response (6 genes), response to bacterium (5 genes), antibacterial humoral response (4 genes), toll signaling pathway (3 genes) and response to fungus (2 genes) (Fig 2). Thus, significant part of up-regulated in LB-flies genes overrepresented GO terms relate to activation of the immune system process (19.4%, p-value < 0.01) and response to stimulus (45.2%, p-value < 0.01), that is a consequence of a violation of cellular homeostasis.

**Fig 3:**
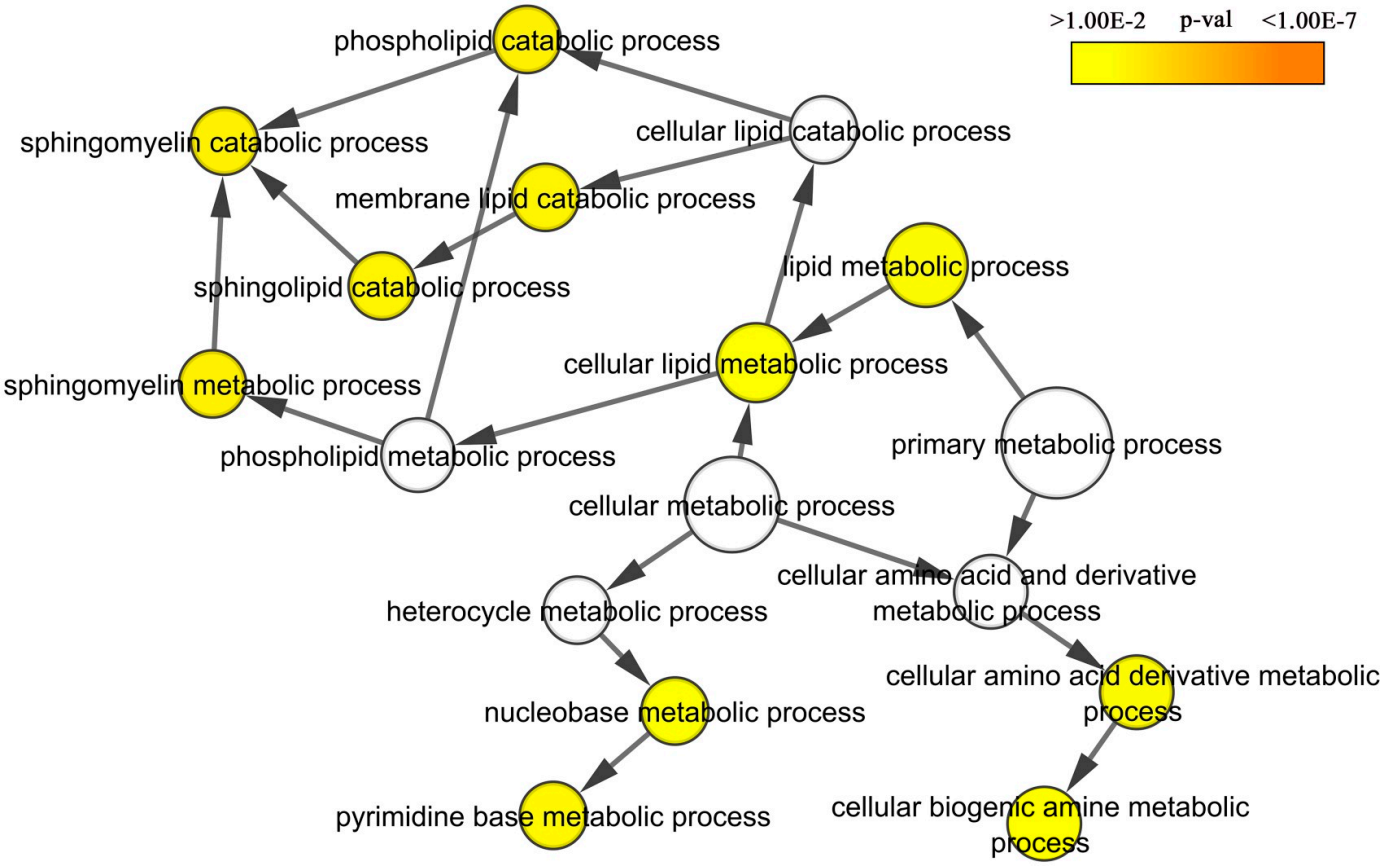
Gene ontology biological process term enrichment analysis for down-regulated LB-flies genes.

Five GO terms were overrepresented for down-regulated in the LB-flies genes with p-value < 0.05: sphingomyelin catabolic process (2 genes), ’de novo’ IMP biosynthetic process (2 genes), mannose metabolic process (2 genes), protein deglycosylation (2 genes) and proteolysis (5 genes). All these processes can be roughly combined into a group of cellular metabolism (56.8% of the down-regulated in the LB-flies genes, p-value < 0.05) (Fig 3).

The significant part of differentially expressed LB-flies genes can be divided into three large categories: cellular metabolism (down-regulated), immune system process and response to biotic stimulus (both up-regulated), which is very similar to the response to some kind of stress factors affecting the LB-flies. In addition, it should be noted that the list of genes that have changed their expression in the LB-flies does not include genes involved in DNA repair, DNA replication, response to oxidative stress, signaling pathways in response to DNA damage, chromatin assembly or disassembly, nucleosome assembly which previously were associated with DNA damage response [41, 42].

A number of genes not included in the DAVID analysis results also need to be considered. Genes *Vmat*, *nAChRbeta1*, *tutl*, *hll*, *Shmtm*, taking part in neural signal transmission (*Vmat*, *Shmt* and *hll* additionally involved in regulation of circadian rhythm), were down-regulated in the LB-flies; *MtnD* gene, strongly inducible by copper, cadmium and other metal ions coding a metallothionein (which is important for metal ion homeostasis and detoxification), was up-regulated in the LB-flies; two genes *CG13075* and *CG14957* predicted to be involved in chitin metabolism were up-regulated in the LB-flies [43–50].

In addition, it should be noted that 16 out of 77 differentially expressed genes had unknown functions, and this fact does not allow making an assumption about their participation in the development of the response to the proposed experimental conditions.

**Fig 4:**
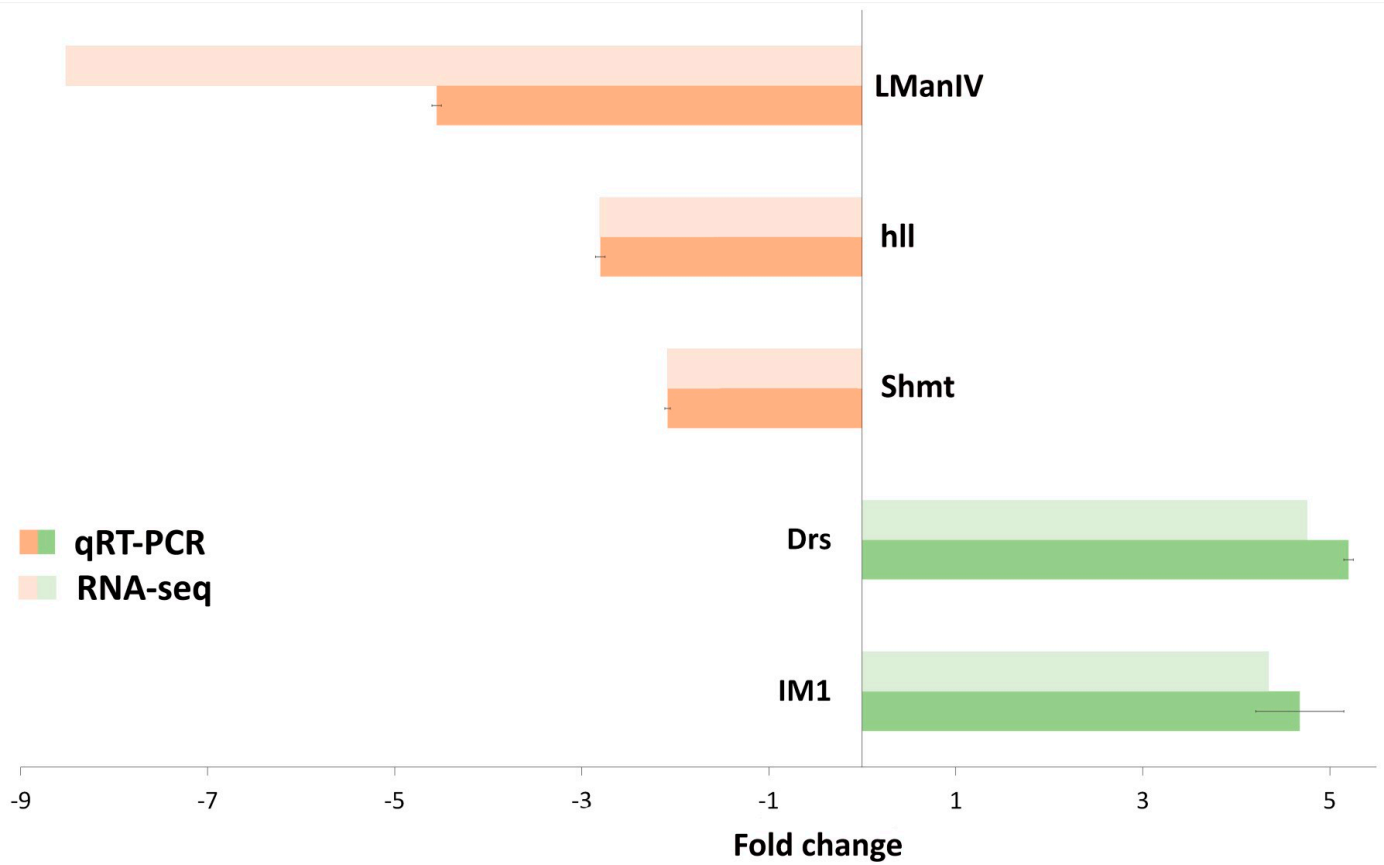
Validation of the RNA-seq results by the RT-qPCR analysis. The bar graphs represent the fold change in gene expression in the LB-flies vs. NB-flies. For all values p < 0.05.

To validate the RNA-seq results we used Real-Time PCR for five differentially expressed genes involved in different important pathways: the defense response, regulation of circadian rhythm and metabolic process (*LManIV*, *hll*, *Shmt*, *Drs*, *IM1*). The results obtained by quantitative RT-PCR agree with the results obtained by RNA-seq, which suggests the reproducibility of the difference in gene expression between the LB- and NB-flies (Fig 4).

## Discussion

In this work, the contribution of low-radiation background to life processes of a model genetic object *D*. *melanogaster* has been evaluated. *D*. *melanogaster* had been already efficiently used for biological studies as an animal model for non-human natural background dosimetry [20, 51]. Additionally, it was shown that the fruit fly is a suitable organism for studies in deep underground low-background laboratories for which the effect of low-radiation background was observed after two weeks of exposition [6]. The input of low background was estimated for the first time for this multicellular complex organism by comparing the results of RNA-seq for flies developed in natural radiation background and in low background conditions. It is important to note that at the moment there is no consensus about the effect of low-radiation background on living organisms—in some experiments the absence of its influence was recorded [13, 16], in others the effect of background radiation reduction was observed [6, 12, 14, 24]. But biological parameters evaluated in different experiments for various model organisms were so diverse and multidirectional that they do not allow making any general conclusion [11, 20, 52]. However, transcriptome analysis of both control and experimental organisms makes it possible to obtain information about changes in all biological processes at once. The use of the complexly organized model organism *D*. *melanogaster* with well studied genetics of many traits [53, 54] simplifies benchmarking.

Natural background radiation consists of terrestrial and cosmic components. That is why the study of low-background radiation effects on biological objects requires controlling many components, such as gamma rays, neutron fluxes, radon concentration, contribution of natural isotopes from the nutrient medium etc. Deep underground low-background laboratories with additional shielding from radiation and equipped with ventilation systems provide the most efficient decrease in natural background radiation [19, 23]. Our low-background radiation experiments were carried out in the DULB-4900 laboratory which is one of the most appropriate locations considering natural radiation protection [19, 32]. In addition, there is a surface laboratory for natural background experiments in the same location near the entrance into the underground laboratory, which guarantees the constancy of a number of environmental parameters for the LB- and NB-flies (for instance, atmospheric pressure). Thus, taking into consideration low contamination of used nutrient medium with naturally occurring radioisotopes and good shielding properties of DULB-4900, we achieved an ~10-fold reduction in natural background radiation, which is similar to the total radiation background reduction by 4–15 times reached in other low-background biological experiments [11, 13, 22].

The analysis of RNA-seq data indicated that expression levels for the most genes between the LB-flies and NB-flies were very similar. We revealed that only 0.44% of the total transcripts (77 out of 17 674) at FDR<0.05 and a fold change value more than 1.5 were significantly altered in the LB-flies developed in the low-background laboratory. This reflected a relatively small adaptive response of organisms to the conditions of DULB-4900. For comparison, the number of differentially expressed genes after fungal treatment at a dose of 10 CFU was 268, and after irradiation at a dose of 20 cGy, it was 380 [55, 56].

**Fig 5:**
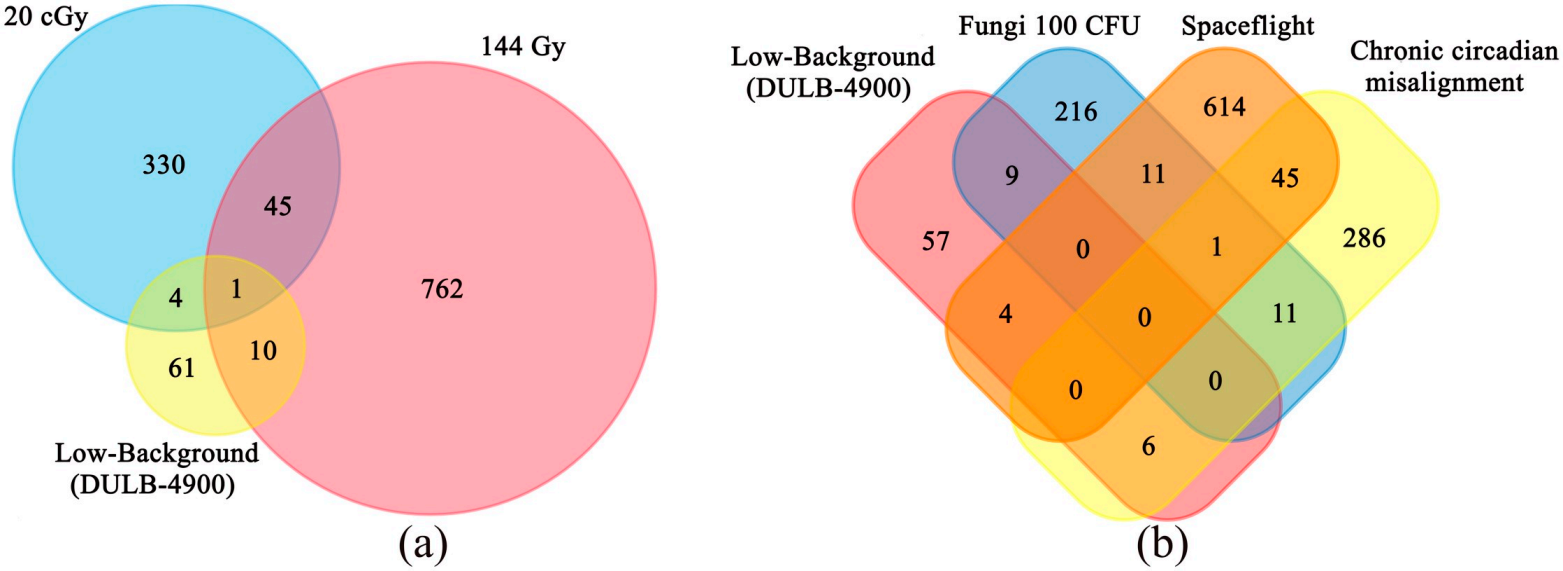
Diagram representing the quantity of shared genes for *D*. *melanogaster* developed in the low-background conditions of DULB-4900 and after different treatments: Irradiation with low (20 cGy), high (144 Gy) doses of ionizing radiation (a) and fungal treatment, spaceflight, chronic circadian misalignment (b).

Since *D*. *melanogaster* was often used as an important model object for radiobiological studies, we had the opportunity to compare our results with transcriptome data obtained in other experiments concerning the effects of different doses of radiation on *D*. *melanogaster*, namely of a high dose (144 Gy with the dose rate of 0.72 Gy min^-1^) and of a low dose (20 cGy with the dose rate of 36 mGy h^-1^) [55, 56] (Fig 5a). All the compared data, as well as ones in our work, were obtained in experiments on the wild type *D*. *melanogaster* males. The only gene that demonstrated alteration in the expression level in all three experiments is *CG12766* whose predicted function is NAD(P)(H) dependent aldo-keto reductase (oxidation-reduction process). Differentially expressed genes, common for the LB-flies and the flies after 20cGy irradiation, were related to cellular metabolism processes (*Hex-C* (decrease in both experiments), *CG18609* (up-regulated in the LB-flies and down-regulated in the 20 cGy flies)), immune response (*Drs*–up-regulated in the LB-flies and down-regulated in the 20 cGy flies) and predicted endonuclease activity (*CG14120*—decrease in both experiments). Differentially expressed genes were associated for the LB-flies and the flies after 144Gy irradiation with cellular metabolism processes (*CG14219*, *CG8834*, *CG18179*, *alpha-Est1*), defense response (*CG43055*, *CG15065*), transmembrane transport (*CG10960*) and cold acclimation (*Fst*). It is important that changes in the activity of metabolic processes, usually indicating the general stress response, were observed when *D*. *melanogaster* were exposed to different types of stress with different intensities [56]. Thus, the comparison of genes that changed their expression in response to a low dose, a high dose and low-radiation background demonstrated that some kind of stress response was observed in low-background conditions. However, this stress quite possible was not specific to radiation scenarios the main distinguishing features of which are response to damage of macromolecular structures (main characteristic of exposure to high doses of radiation) or processes associated with an increase of ROS amount (main effect after low dose radiation treatment) [57–59].

The linear no-threshold (LNT) model postulates a positive linear correlation between an absorbed dose of radiation and cell damage. Based on this theory, most hypothesis concerning influence of low-radiation background on organism predicted that decrease of radiation background would cause reduction of radiation-induced reactive oxygen species (ROS) which in turn would cause changes in the work of a number of cell systems (for example, signaling pathways, DNA maintaining systems etc.) [3, 24, 27]. If we assume that some biological processes occur depending on the radiation level, then these processes under conditions of low-radiation background and irradiation should show opposite values. Therefore, we compared the up-regulated biological processes in the *D*. *melanogaster* wild type males after irradiation (144 Gy and 20 cGy) [55, 56] with down-regulated processes in the LB-flies and saw nothing in common in the enriched biological processes between the LB-flies and the 20 cGy irradiated flies and one common process between the LB-flies and the 144 Gy irradiated flies—the oxidation-reduction process (GO:0055114). However, differentially expressed genes in the LB-flies which took part in the “oxidation-reduction process” presented both in up- and down-regulated categories, therefore, it cannot be suggested that this biological process is reduced under conditions of low-radiation background. Thus, we did not observe changes in expression of genes that are used as biomarkers of radiation induced stress in the LB-flies neither during the analysis of individual genes nor considering biological processes. It should be noted that our data agree with the simulation data showing that the dose rate equal to 417 μGy h^-1^ and lower did not significantly impact ROS concentration and cellular redox potential in cells [60]. Thus, the data obtained in our experiment seem not to agree with the linear no-threshold (LNT) risk model assuming extrapolation of the effects of high-radiation doses to the area of very low-radiation doses with no safety threshold and DNA damage linearly proportional to the dose [8, 61]. Wadsworth et al. came to similar conclusions after researching the impact of the Boulby Underground Laboratory conditions on bacterial models [16]. Based on these data, it can be assumed that below a certain level of background radiation the stress caused by radiation in *D*. *melanogaster* becomes negligible, in our case, this level is 16.4 nGy h^-1^.

This leads to the assumption that the response of *D*. *melanogaster* to the conditions of DULB-4900 may reflect adaptation not to low-radiation background only, but to some other deep underground environmental parameters, and the effect of low background on radiation dependent processes was probably either absent at all or so insignificant that it was below the level of detection by transcriptome analysis. To explain what could have caused the observed changes in gene expression in the LB-flies, we compared our data with the data of transcriptome analysis of *D*. *melanogaster*, exposed to different stress conditions, deposited in the GEO Database and other publicly available datasets (Fig 5b). The largest number of genes differentially expressed in the LB-flies was common with differentially expressed genes after fungal treatment: *Drs*, *Fst*, *IM1*, *IM2*, *IM3*, *IM23*, *Spn88Eb*, *CG15065*, *CG18179* [56]. All listed genes (except *CG18179*) and additionally two genes involved in immune response *GNBP-like3* and *Tep2* were up-regulated in the LB-flies. It is likely that the activation of these genes in the LB-flies may be the result of exposure to the microbiome of the underground laboratory which is of course different from the microbiome of the surface laboratory conditions and appears to be unusual or more aggressive to *D*. *melanogaster*.

Six genes with altered expression coincided for the LB-flies and *D*. *melanogaster* with chronic circadian misalignment: *MtnD*, *Ser8*, *CG13905*, *CG18609*, *CG10960*, *CG34136* [44]. Additionally, between genes, differentially expressed in the LB-flies, there were several genes with the proven participation in regulation of sleep and circadian rhythms–*hll*, *Shmt* and *Vmat* [45–47] (all down-regulated in the LB-flies). Apparently, it indicates a disturbance in sleep and circadian rhythms in the LB-flies. It seems that the likely cause of these disturbances may be a lack of natural external stimuli underground, in particular sounds, odors and vibrations, essential components of normal living conditions for terrestrial organisms. For instance, vibration stimuli contribute to the establishment of the *D*. *melanogaster* circadian clock through chordotonal organs stimulation [62]. This hypothesis is also supported by decrease in expression of the *tutl* gene in the LB-flies. The activity of this gene is associated with signals from chordotonal organs [50].

It is interesting that staying on the space station (SpaceX-5 mission, absorbed dose rate 8300 nGy h^-1^, GLDS-278 [63]) caused similar reactions in flies only to a greater extent– 45 genes with altered expression were common for chronic circadian misalignment and the effects of space station conditions [44, 64]. Additionally, the LB-flies and the space station flies also had some common differentially expressed genes: *GNBP-like3* (detection of biotic stimuli; response to fungus; defense response to other organisms [65, 66]), *Tsp42Ecn* (unknown functions), *mino* (encoding glycerol-3-phosphate O-acyltransferase, involved in fatty acid metabolic process and piRNA biogenesis [67]), *CG7542* (predicted serine-type endopeptidase). Comparison data between LB-flies with an earlier data from 12 days space flight Oregon-R males (NASA Space Shuttle Discovery STS-121, absorbed dose rate 9200 nGy h^-1^) [68] revealed three common differentially genes: *Fst* (involved in cold acclimation [69]), *CG10337*, *CG10182*. Thus, genes related to multi-component stress response to space flight environments (including chronic elevated radiation background and microgravity [70]) almost did not overlap with genes involved in adaptive response to DULB-4900 conditions.

It should be noted that the changes in expression of some genes in the LB-flies can be interpreted as suppression of nerve impulse transmission—*nAChRbeta1* (predicted acetylcholine-gated cation-selective channel activity), *Vmat* (coding protein that repackages monoamines (dopamine, serotonin, and octopamine)) into presynaptic vesicles [48]) and *tutl* (coding transmembrane protein involved in coordinated motor control [49, 50])—all down-regulated in the LB-flies.

Additional KEGG pathway analysis of differentially expressed genes for LB-flies revealed that they were enriched in “Metabolic pathways”, “Biosynthesis of antibiotics” and “Other glycan degradation” (S2 Table). The only experiment where we can find the DEGs involved in the same KEGG pathways (“Metabolic pathways” and “Biosynthesis of antibiotics”) was fungal treatment [56]. Looking at the results of all comparisons from the different sides of enrichment analysis together, we can conclude that the closest set of genes with altered expression to LB-flies DEGs was the set after fungal treatment.

As noted earlier, in present there is no single point of view about whether a decrease in background radiation affects living organisms. In several studies a stress response registered in organisms exposed to below background radiation was explained by the absence of some usual level of environmental radiation [14, 20, 27]. That means the existence of some hormetic effects arising in the presence of radiation background that may be important to maintain optimal homeostasis of living systems. On the other hand, several studies have revealed the inability to change growth and development parameters of living organisms in low background radiation conditions and assumed an existence of a threshold for radiosensitivity [3, 13, 16].

We believe that considering the effects of low-radiation background in the deep underground laboratories, it is necessary to take into account one more factor that is impossible to control—the influence of deep underground conditions that are not normal for all multicellular terrestrial model organisms and require some adaptation to them. Thus, responses of complex organisms to conditions of deep underground low-radiation background laboratories probably more correct to consider from two points of view—as an effect of decrease in radiation background and as the influence of deep underground conditions. It is important that analyzing our data, we faced the lack of information about effects of deep underground conditions on complex multicellular organisms. Timing of hatch, percent survival and several morphometric parameters of the lake whitefish (*C*. *clupeaformis*) were studied in the SNOLAB pilot experiment [15]. Additionally, Moricano et al. estimated several physiological parameters of *D*. *melanogaster* (lifespan, fertility etc.) [20], but this is definitely not enough to understand the overall picture of effects of underground laboratory environments. We hypothesize that a number of changes in gene expression was caused in our experiment by the lack of a necessary level of external stimuli in underground conditions, such as sounds and vibrations, which could have triggered circadian rhythm disturbances and subsequent deterioration in functioning of some neuromuscular system components. Therefore, our results may also be important in terms of adaptation of multicellular organisms to deep underground conditions and for simulation of underground exoplanetary conditions for further space explorations [71–73]. It is interesting to note that we did not observe differentially expressed genes involved in hypoxia between the LB and NB experimental flies. Such genes were commonly registered during a biological response to industrial deep underground or cave conditions [74]. This fact can indicate a high quality of the ventilation system in DULB-4900.

As limitations of our study, we should note that this is the first pilot work and the biological experiment in the DULB-4900 was carried out for the first time and once. Therefore, further experiments investigating different time points of *D*.*melanogaster* life cycle are required and our data should be considered as the basis for hypothesis explaining the observed changes in gene expression in LB-flies.

In conclusion, our results showed a very limited *D*. *melanogaster* response to the deep underground environment resulted in a relatively small amount of differentially expressed genes that are not specific for radiation related pathways. In part, this response may be caused by the lack of some physical stimuli affecting organisms on the surface, including the possible influence of reduced background radiation which almost does not overlap with the well-studied effect of low and high doses of radiation. Observed changes in gene expression may reflect an adaptive response to underground conditions of DULB-4900 and appear to suggest the presence of a certain dose threshold below which no common harmful effects of radiation are observed.

## Supporting information

S1 Fig Electrophoregram of RNA samples used for sequencing. Lines: 1 –molecular ruler, 2,3 –NB samples, 3,4,5 –LB samples.
(TIF)

S2 Fig Correlation map for gene expression between repeats of NB- and LB-samples. (TIF)

S1 Table List of primers used for RT-qPCR. (PDF)

S2 Table Altered KEGG pathways for different *D*. *melanogaster* experiments. (PDF)

S1 Raw image (PDF)
