# Supplementary figures and images for: First transcriptome profiling of D. melanogaster after development in a deep underground low radiation background laboratory
Source: PLoS One. 2021 Aug 5;16(8):e0255066. doi: 10.1371/journal.pone.0255066 (PMC8341612; doi:10.1371/journal.pone.0255066)

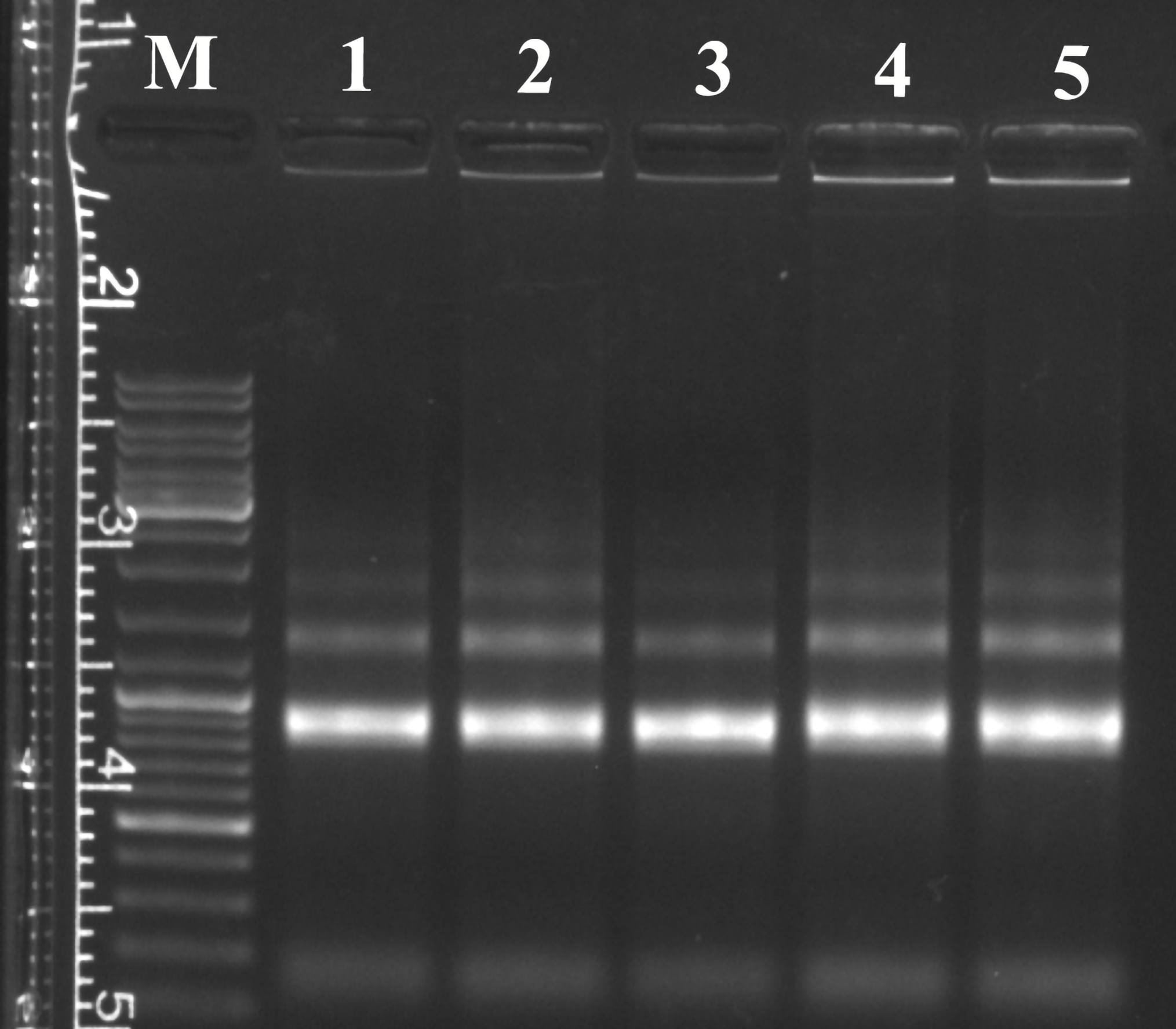

Supplement: S1 Fig — Lines: 1 –molecular ruler, 2,3 –NB samples, 3,4,5 –LB samples. (TIF) [file pone.0255066.s001.tif]

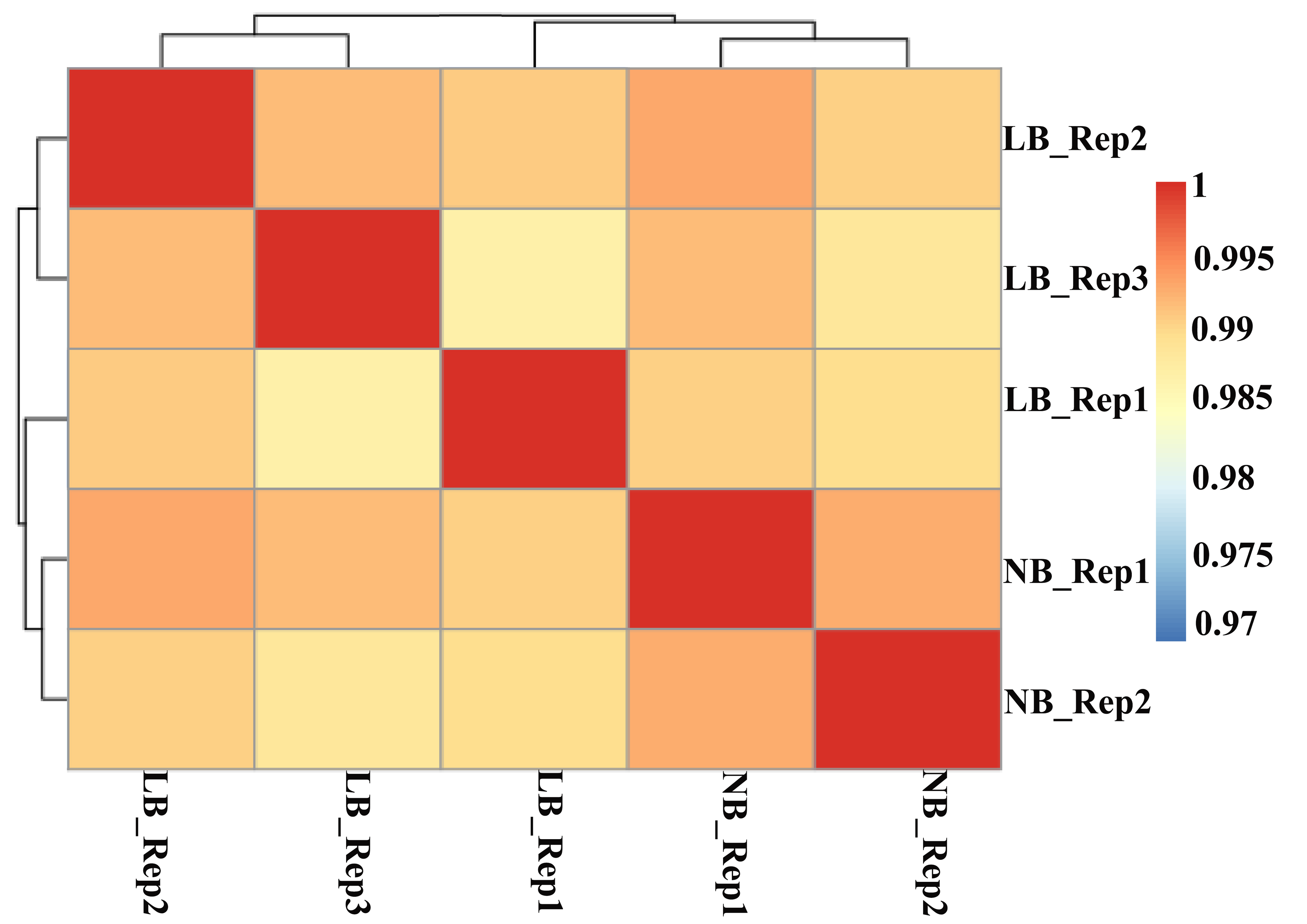

Supplement: S2 Fig — (TIF) [file pone.0255066.s002.tif]
