# Supplementary material for: First transcriptome profiling of D. melanogaster after development in a deep underground low radiation background laboratory
Source: PLoS One. 2021 Aug 5;16(8):e0255066. doi: 10.1371/journal.pone.0255066 (PMC8341612; doi:10.1371/journal.pone.0255066)
Supplement: S1 Table — (PDF) [file pone.0255066.s003.pdf]

S1 Table. List of primers used for RT-qPCR

IM1-rt-Fw	CCAATGCTGTTCCACTGTCG
IM1-rt-rev	CGAATCCTTGGGTTGAAACTTCC
Shmt-rt-Fw	GCGGTGTTTCCATCACTCCA
Shmt-rt-rev	AGGGCCTTGGCATTCTTGAG
Hll-rt-Fw	GAACGACCTCAAGCCAGCTAC
Hll-rt-rev	GGGCAGATCCGAATACTTCTCG
LManIV-rt-Fw	GGATGGTGCCCTAGAGATGC
LManIV-rt-rev	TCGCGGAATCGAGAATGAGG
Drs-rt-Fw	CCCTCTTCGCTGTCCTGATG
Drs-rt-rev	CTCCTTGCACACACGACGA
Rpl32-rt-Fw	GTTCGATCCGTAACCGATGTTG
Rpl32-rt-rev	CCAGTCGGATCGATATGCTAAG
