# Supplementary material for: First transcriptome profiling of D. melanogaster after development in a deep underground low radiation background laboratory
Source: PLoS One. 2021 Aug 5;16(8):e0255066. doi: 10.1371/journal.pone.0255066 (PMC8341612; doi:10.1371/journal.pone.0255066)
Supplement: S2 Table — (PDF) [file pone.0255066.s004.pdf]

S2 Table. Altered KEGG pathways for different *D. melanogaster* experiments

KEGG Pathway						
Metabolic pathways	10				33	
Biosynthesis of antibiotics	4				11	
Other glycan degradation	2					
Glutathione metabolism		10	5			7
Drug metabolism - cytochrome P450		9	6			6
Metabolism of xenobiotics by cytochrome P450		9	6		4	7
Starch and sucrose metabolism		5			5	
Galactose metabolism		5			6	
Drug metabolism - other enzymes		5				
Wnt signaling pathway			10	10		
Non-homologous end-joining			3			
Ribosome biogenesis In eukaryotes			8			
Ascorbate and aldarate metabolism			3			
Retinol metabolism			3			
TGF-beta signaling pathway			3			
Porphyrin and chlorophyll metabolism			3			
Pentose and glucuronate interconversions			3			
Hedgehog signaling pathway				4		
RNA degradation				7		
Circadian rhythm - fly				3		
Hippo signaling pathway - fly				6		
Jak-STAT signaling pathway				3		
mRNA surveillance pathway				6		
FoxO signaling pathway				5		
Pentose and glucuronate interconversions					8	
2-Oxocarboxylic acid metabolism					3	
Glycerolipid metabolism					4	
Glycine, serine and threonine metabolism					3	
Biosynthesis of amino acids					4	
Ascorbate and aldarate metabolism					3	
Carbon metabolism					5	
Purine metabolism						9
Endocytosis						8
	DULB-4900	40 cGy (Moskalev et al 2014)	144 Gy (Moskalev et al 2015)	Cyrcadian rhythm misalignment (Boomgarden et al 2019)	Fungus (Moskalev et al 2015)	Spaceflight (Taylor et al 2014)
