# Supplementary material for: First transcriptome profiling of D. melanogaster after development in a deep underground low radiation background laboratory
Source: PLoS One. 2021 Aug 5;16(8):e0255066. doi: 10.1371/journal.pone.0255066 (PMC8341612; doi:10.1371/journal.pone.0255066)
Supplement: S1 Raw image — (PDF) [file pone.0255066.s005.pdf]

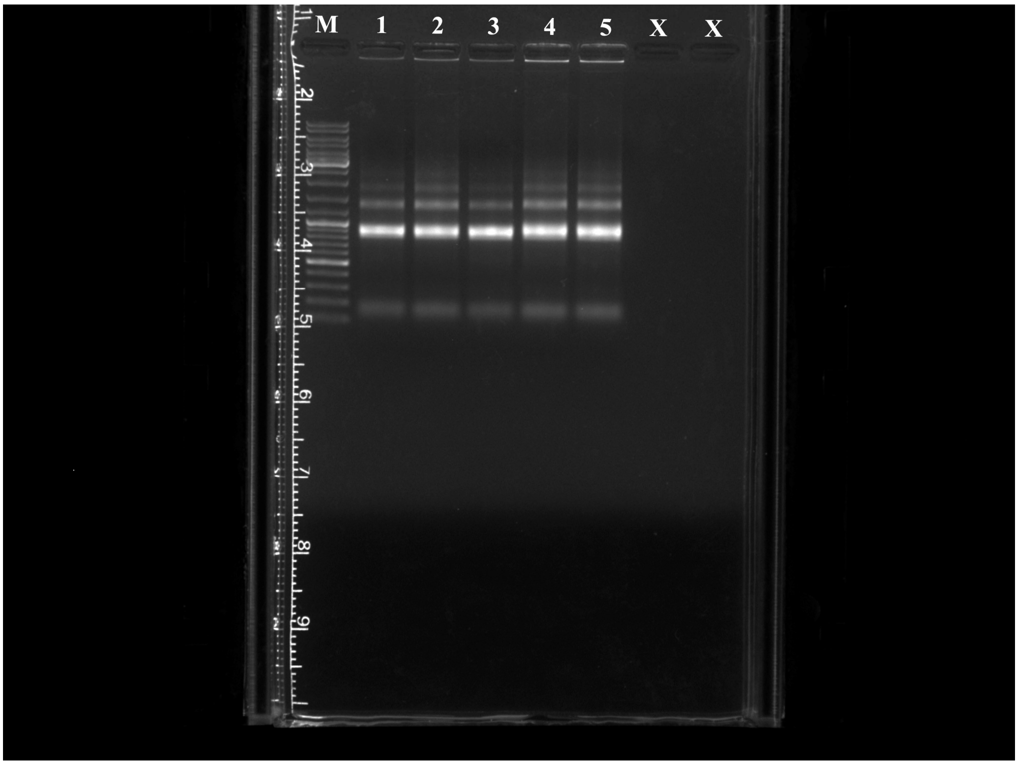

S1 Figure. Electrophoregram of RNA samples used for sequencing. Lines: M – molecular weight marker, 1,2 – NB samples, 3,4,5 – LB samples.
